# Cerebrospinal Fluid Matrix Metalloproteinases Are Elevated in Cerebral Adrenoleukodystrophy and Correlate with MRI Severity and Neurologic Dysfunction

**DOI:** 10.1371/journal.pone.0050430

**Published:** 2012-11-21

**Authors:** Kathryn A. Thibert, Gerald V. Raymond, David R. Nascene, Weston P. Miller, Jakub Tolar, Paul J. Orchard, Troy C. Lund

**Affiliations:** 1 Division of Pediatric Blood and Marrow Transplantation, University of Minnesota, Minneapolis, Minnesota, United States of America; 2 Department of Neurogenetics, Kennedy Krieger Institute, Baltimore, Maryland, United States of America; 3 Department of Radiology, University of Minnesota, Minneapolis, Minnesota, United States of America; Biological Research Centre of the Hungarian Academy of Sciences, Hungary

## Abstract

**Background:**

X-linked adrenoleukodystrophy results from mutations in the *ABCD1* gene disrupting the metabolism of very-long-chain fatty acids. The most serious form of ALD, cerebral adrenoleukodystrophy (cALD), causes neuroinflammation and demyelination. Neuroimaging in cALD shows inflammatory changes and indicates blood-brain-barrier (BBB) disruption. We hypothesize that disruption may occur through the degradation of the extracellular matrix defining the BBB by matrix metalloproteinases (MMPs). MMPs have not been evaluated in the setting of cALD.

**Methodology/Principal Findings:**

We used a multiplex assay to correlate the concentration of MMPs in cerebrospinal fluid and plasma to the severity of brain inflammation as determined by the ALD MRI (Loes) score and the neurologic function score. There were significant elevations of MMP2, MMP9, MMP10, TIMP1, and total protein in the CSF of boys with cALD compared to controls. Levels of MMP10, TIMP1, and total protein in CSF showed significant correlation [p<0.05 for each with pre-transplant MRI Loes Loes scores (R^2^ = 0.34, 0.20, 0.55 respectively). Levels of TIMP1 and total protein in CSF significantly correlated with pre-transplant neurologic functional scores (R^2^ = 0.22 and 0.48 respectively), and levels of MMP10 and total protein in CSF significantly correlated with one-year post-transplant functional scores (R^2^ = 0.38 and 0.69). There was a significant elevation of MMP9 levels in plasma compared to control, but did not correlate with the MRI or neurologic function scores.

**Conclusions/Significance:**

MMPs were found to be elevated in the CSF of boys with cALD and may mechanistically contribute to the breakdown of the blood-brain-barrier. MMP concentrations directly correlate to radiographic and clinical neurologic severity. Interestingly, increased total protein levels showed superior correlation to MRI score and neurologic function score before and at one year after transplant.

## Introduction

X-linked adrenoleukodystrophy (ALD) is a neurometabolic disease that results from mutations in *ABCD1*, the gene that encodes for a peroxisomal transporter of very long chain fatty acid (VLCFA) and subsequently disrupts their metabolism and results in the accumulation of these compounds in all tissues. [1]. ALD predominantly affects the adrenal cortex, testes, and nervous system. The nervous system manifestations are variable. Cerebral disease is the most common neurologic presentation in childhood. Adrenomyeloneuropathy is an adult presentation and is characterized by involvement of the long tracts of the spinal cord. However, adult men may also develop cerebral disease. It is important to point out that while ALD is characterized by the accumulation of VLCFA, only 35% of individuals at risk will develop cerebral disease in childhood and no correlation has been observed between the accumulation of the fatty acids in serum and the clinical phenotype for ALD [1]. There is also no known correlation between mutation and phenotypes, making it difficult to predict the presentation or progression of the disease.

Cerebral ALD is characterized by central inflammatory demyelination [2]. The five-year survival rate after first appearance of clinical symptoms of cerebral inflammation is 59% [3]. In boys with an *ABCD1* mutation, there is currently no reliable way to predict who will develop neuroinflammation or when neuroinflammation may occur. There are very few therapeutic options once neuroinflammation occurs. Treatment with Lorenzo’s oil (glycerol trioleate and glycerol trierucate) combined with a VLCFA-low diet normalizes the concentration of VLCFA and may delay neurologic disease in those predestined to develop it, but it does not slow the progress of neurological symptoms once they have begun [4]. Hematopoietic cell transplant (HCT) is the only way to reduce the cerebral inflammation and arrest the demyelination process once it has started [2], although the mechanism underlying the disease attenuation remains unknown. What is clear is that a distinction can made between the long-term survival rate of patients receiving HSCT in the early-stages of cerebral inflammation versus those in later stages, thus indicating a need for a method to earlier identify boys who will develop cALD [2]. Identification of these boys would provide an opportunity to intervene in the neuroinflammatory process earlier leading to a superior clinical outcome.

A defining feature found on the MRIs of boys with cALD is the enhancement around the apparent lesion upon infusion of gadolinium indicating an ongoing pathological and neuroinflammatory process. While this is true in any neuroinflammatory disorder, the garland of enhancement of a posterior white matter lesion in a boy is nearly pathognomic of ALD [1]. This enhancement is consistent with disruption of the blood brain barrier (BBB) allowing entry of inflammatory cells. A proposed mechanism of BBB disruption could be by the increased expression of matrix metalloproteinases (MMPs), which degrade the extracellular matrix defining the BBB capillary network. This is accompanied by the invasion of lymphocytes or monocytes accompanied by cytokine secretion, cytokine receptor shedding, and cell-mediated damage [5]–[6]. Tissue inhibitors of metalloproteinases (TIMPs) are directly responsible for inhibiting MMPs may also be involved in this inflammatory process as they maintain the balance between deposition and degradation in the extracellular matrix. Levels of MMPs and TIMPs have not previously been explored in the context of cALD.

In preparation for HCT, we have collected CSF and plasma samples from boys with cALD prior to their undergoing transplant and have previously reported elevated cytokine levels that correlated to the ALD MRI score [7], [8]. The objectives of this study were: (1) to evaluate the concentration of MMPs and TIMPs in CSF and plasma for cALD boys as compared to controls, and (2) to correlate the concentration of MMPs and TIMPs to the severity of brain inflammation as determined by MRI in boys with cALD based on the Loes scoring system.

## Methods

### Ethics

This study and the use of all bodily fluids were approved by the Committee on the Use of Human Subjects in Research at the University of Minnesota. Informed written consent was obtained for all patient samples from the parents or guardians on behalf of the child participants. Patient written assent was also obtained if patients were greater than 8 years of age.

### Participants

Samples were taken from patients with cALD (n = 20) 2 to 6 months prior to HSCT at the University of Minnesota. Controls (*n* = 19) were patients undergoing intrathecal chemotherapy as treatment for a prior diagnosis of acute lymphoblastic leukemia (ALL) and were at least 3 months into maintenance therapy and without CSF leukemia. These patients were deemed to be the most suitable control group because the risk of obtaining CSF from “healthy” controls was too great. Use of these patients as controls has been previously published [7], [8].

### Description of Procedures

Boys in this cohort had been referred to the University of Minnesota Division of Pediatric Blood and Marrow Transplantation as candidates for HSCT. Neurologic function scores of the boys were determined prior to transplant according to the scoring system in Table 1. During a sedated pre–HSCT MRI, a lumbar puncture was performed and 3 milliliters of CSF was obtained and analyzed for protein level, cell count, and MMP and TIMP analysis. Plasma was also collected and analyzed similarly. The same procedure was used for control samples except an MRI was not performed for children in ALL maintenance therapy.

**Table 1:** Neurologic Function Score for boys with cALD.

Hearing/auditory processing problem	1
Aphasia/apraxia	1
Loss of communication	3
Vision impairment/fields cut	1
Cortical blindness	2
Swallowing difficulty or other central nervous dysfunction	2
Tube feeding	2
Running difficulties/hyper-reflexia	1
Walking difficulties/spasticity/spastic gait (no assistance)	1
Spastic gait (needs assistance)	2
Wheelchair required	2
No voluntary movement	3
Episodes of incontinency	1
Total incontinency	2
Presence of nonfebrile seizures	1
**Possible Total**	**25**

This table indicates how neurologic function scores are determined in boys with cALD. Children without dysfunction start with a score of 0.

### Statistical Methods

Measurement of metalloproteinases was performed using a multiplex enzyme-linked immunosorbent assay (ELISA) for a panel of human MMPs including MMP-1, −2, −3, −8, −9, −10, −13, TIMP-1, and TIMP-2 (Aushon Biosystems, Billerica, MA). Each sample was run in duplicate and an average value was calculated to determine the MMP or TIMP concentration using standard curves generated with the relevant recombinant human proteins provided with the commercial kits. Means for the cALD and control groups were calculated and subjected to a two-tailed Student’s t-test to determine a p-value. A linear regression analysis of MMPs and TIMPs of interest and MRI Loes score or neurologic function score was performed using GraphPad Prism software (version 5.0d).

## Results

We performed a comprehensive examination of MMP, TIMP, and total protein levels in the CSF of boys with cALD prior to HSCT (n = 20) versus control patients (n = 19). Our approach was to use a multiplex ELISA for a panel of human MMPs including MMP-1, −2, −3, −8, −9, −10, −13, TIMP-1, and TIMP-2. Initial analysis of the data showed no significant differences between our cALD and control groups for: MMP1, MMP3, MMP8, MMP13 or TIMP2 levels in the CSF (data not shown). The MMP family members for which we found differences are reported below and were subjected to a more detailed analysis and clinical correlation.

**Figure 1:**
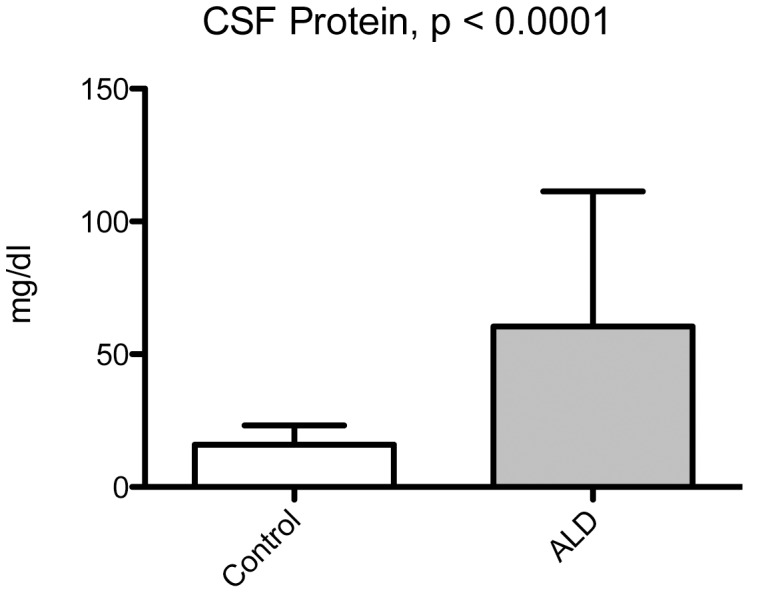
Boys with cALD have elevated CSF protein levels. Total protein levels were determined in the clinical chemistry laboratory using a Kodak Ektachem method. Shown are the means ± S.D. A Student’s t-test was performed to generate the p-value.

**Figure 2:**
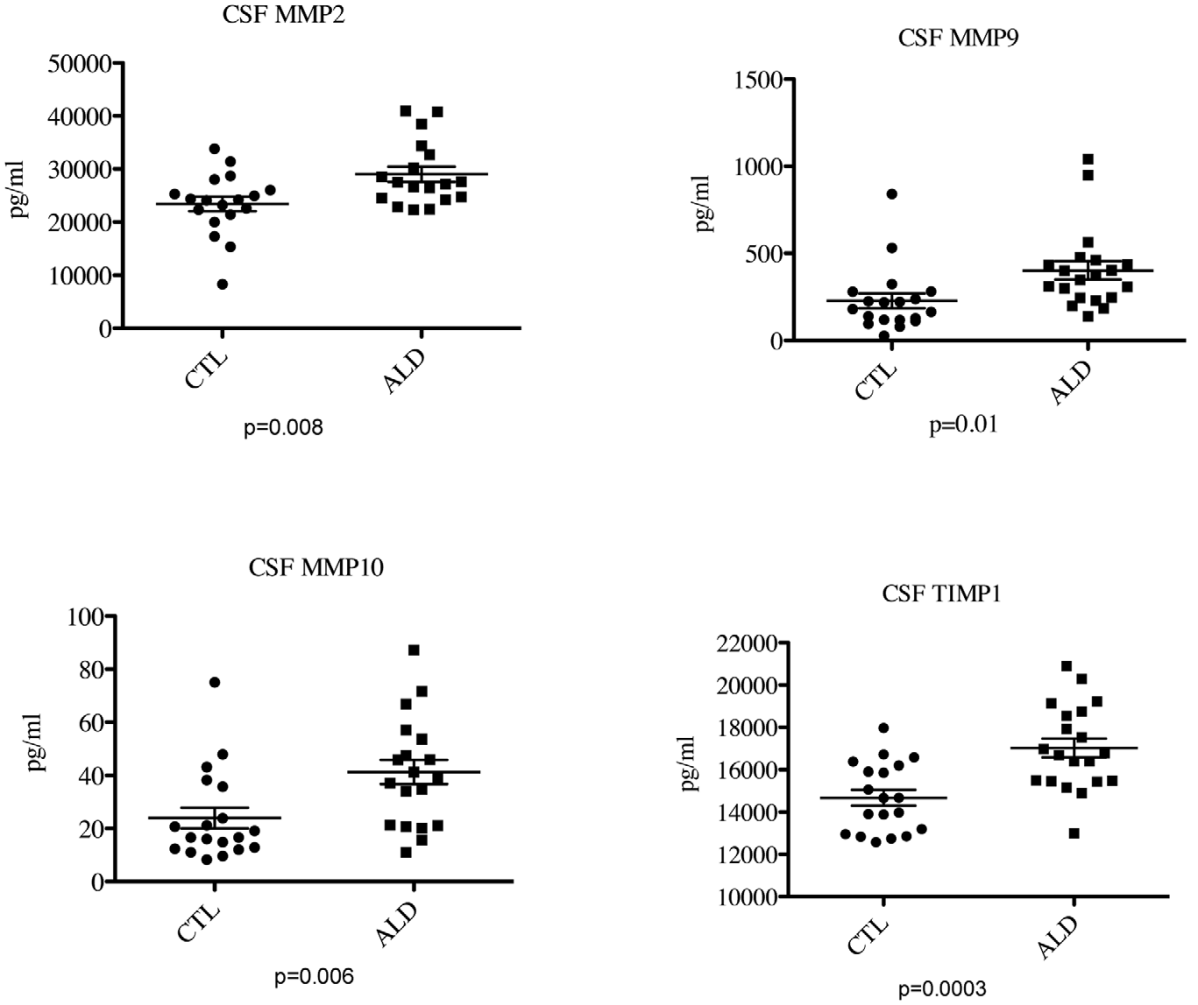
Boys with cALD have higher levels of CSF MMPs and TIMP1 than controls. CSF MMPs and TIMPs were evaluated using a Luminex system. Statistical analysis was performed using Prism software. The data displayed are those MMPs and TIMP1 that were significantly different from the control group. The horizontal bar represents the mean.

The level of total protein in the CSF of boys with cALD was significantly elevated (p<0.0001) as indicated in Figure 1. Analysis of MMP/TIMP family members revealed elevated levels of MMP2 (28090 pg/mL ±1634 vs. 22990 pg/mL ±1256, p = 0.02), MMP9 (402.2 pg/mL ±51.6 vs. 227.8 pg/mL ±42.6, p = 0.01), MMP10 (41.26 pg/mL ±4.496 vs. 24.14 pg/mL ±4.12, p = 0.009), and TIMP1 (17020 pg/mL ±447.3 vs. 14580±385.7 pg/mL, p = 0.0002) in cALD boys versus controls, respectively (Figure 2).

**Figure 3:**
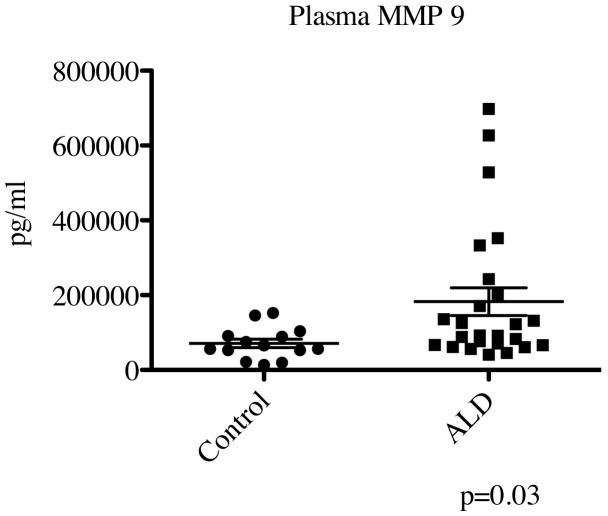
Boys with cALD have higher levels of plasma MMP9 than controls. Plasma MMP9 was evaluated using a Luminex system as before. Statistical analysis was performed using Prism software (version 5.0d). The horizontal bar represents the mean.

We also performed a comprehensive examination of MMP/TIMP family members in the plasma of boys with cALD that showed a significant elevation in MMP9 levels (184847±36958 pg/mL vs. 71179±11343 pg/mL, p = 0.03) as shown in Figure 3. No other MMP or TIMP in our panel showed a significant difference between control and cALD in the plasma (data not shown).

**Figure 4:**
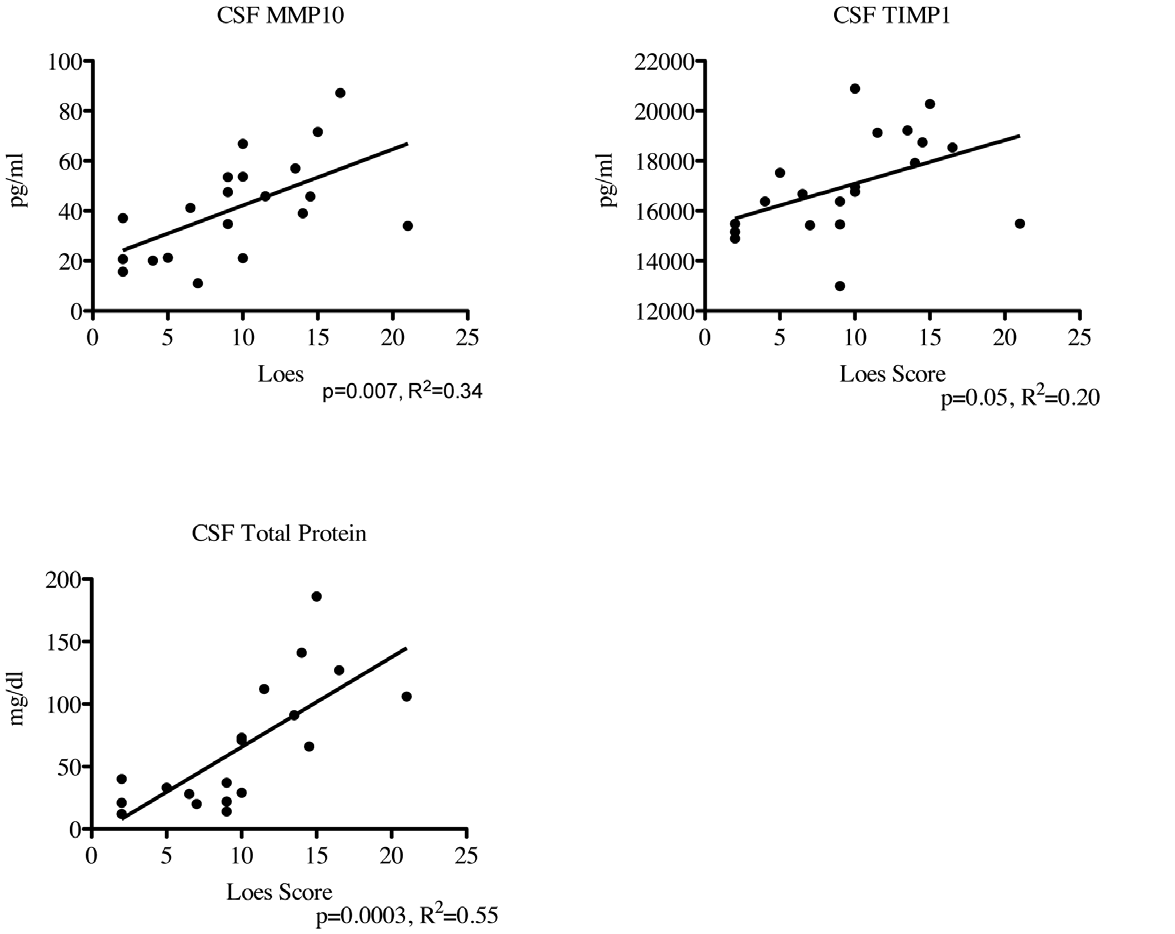
Levels of MMP10, TIMP1, and CSF total protein correlate with pre-HCT Loes MRI Score. Regression analysis was performed using the previously determined MMP and TIMP1 levels and each patient’s pre-transplant Loes score. P-values and R^2^ were determined using Prism (version 5.0d).

The ALD MRI (Loes) score is an MRI-based quantification of the amount of cerebral involvement; it is based on a point system derived from location, extent of disease, and the presence of focal and/or global atrophy that is present in boys with cALD [9]. All cALD patients had MRIs prior to transplant that were scored by a single neuroradiologist. The Loes score correlated significantly with CSF MMP10 (p = 0.007, R^2^ = 0.34) and CSF TIMP1 (p = 0.05, R^2^ = 0.20) as shown in Figure 4. No other MMP or TIMP correlated significantly with the pre-transplant Loes score. Interestingly, CSF total protein levels also correlated significantly with the Loes score having the most significant p-value and best correlation coefficient (p = 0.0003, R^2^ = 0.55).

**Figure 5:**
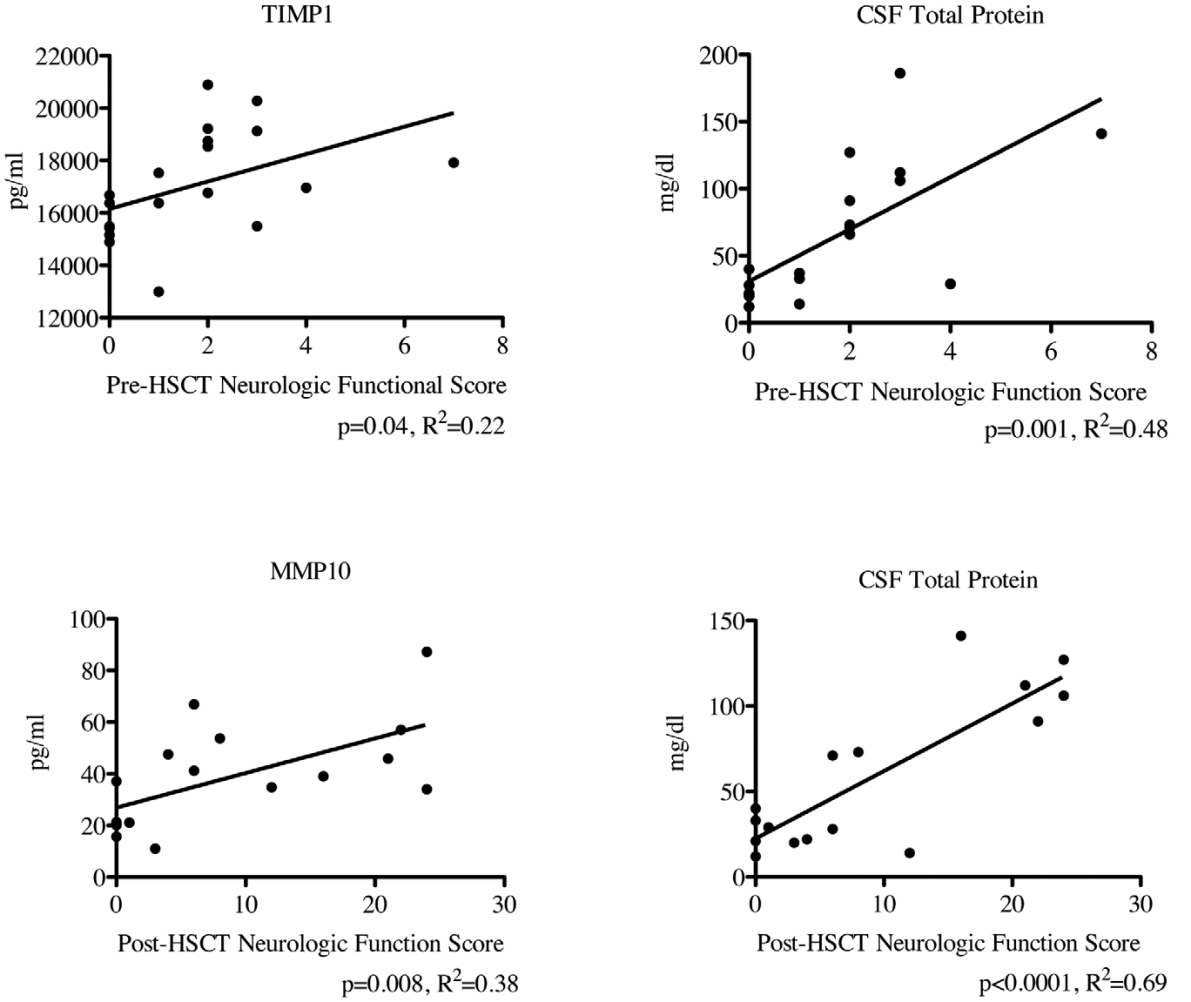
Levels of CSF TIMP, MMP10, and total protein correlate with neurologic score pre- and post-HSCT. Regression analysis was performed using the previously determined MMP and TIMP levels and each patient’s pre- and post-HCT neurologic function score. The post-HSCT scores were determined at the one year post-HCT follow-up visit. P-values and R^2^ were determined using Prism software (version 5.0d).

The neurologic function score quantifies the gross neurological dysfunction through assessment of patients in the following areas: vision, hearing, speech, gait, and other areas such as fine motor skills and activities of daily living as shown in Table 1. For each area that an individual patient has a deficit, points are given. This scoring system has been used in several studies to follow a patient’s “neurologic trajectory” over time and also correlates to the MRI score and CSF chitotriosidase activity [2], [7], [10]. Pre-transplant functional scores correlated significantly with CSF levels of TIMP1 and CSF total protein levels as shown in Figure 5 (p = 0.04, R^2^ = 0.22; and p<0.001, R^2^ = 0.48, respectively). With regard to transplant outcomes, post-transplant neurologic functional score correlated significantly with CSF levels of MMP10 and CSF total protein levels (R^2^ = 0.38, p = 0.008 and R^2^ = 0.69, p<0.0001 respectively). None of the other MMPs and TIMPs in our panel correlated significantly to pre-transplant or post-transplant functional scores.

## Discussion

This study provides the first evidence of elevated MMPs and TIMPs in the CSF of boys with cALD. We report that there are significant elevations of CSF total protein, MMP2, MMP9, MMP10, TIMP1 and plasma MMP9 in boys with cALD at the time of transplant compared to controls. The levels of MMP10 and TIMP1 in CSF correlated significantly with the pre-transplant MRI scores. The levels of TIMP1 and CSF total protein also correlated significantly with the pre-transplant neurologic functional scores, and the levels of MMP10 and CSF total protein correlated significantly with the 1-year post-transplant neurologic functional score.

MMP9 is a fairly well studied metalloproteinase, and increases in MMP9 have been shown in the CSF of patients with both viral and bacterial meningitis [11]. This elevation was found to specifically be associated with parenchymal gelatinolytic activity. MMP9 has also been shown to be elevated in multiple sclerosis (MS) and levels have been correlated to severity of MRI findings correlating with an increased number of gadolinium-enhancing MRI lesions or with the risk of developing new lesions. MMP9 levels were also increased during clinical relapses [12]–[13]. Furthermore, reports have indicated that high CSF MMP9 levels precede the appearance of new enhancing lesions and that corticosteroids reverse the occurrence of the lesions by reducing the MMP9 activity [14]. It has been shown that MMP9 interacts with various cell surface components and that this type of interaction positively regulates enzymatic activation and activity. Experiments using a mouse model have indicated that MMP9 was a main contributor to BBB disruption in focal cerebral ischemia, which substantiates it role in neuropathology [15].

Elevated levels of MMP2 and MMP10 were also observed in boys with cALD, and MMP2 has been shown to be significantly higher in the brain following traumatic or viral brain injury [12]. MMP2 is similar to MMP9 in its specific binding affinity to gelatin [12], and therefore, may play a similar role in the neuroinflammation of cALD. There is much less known about the role of MMP10 MMP10 in neurodegenerative diseases. It is known that when unregulated, increases levels of MMP10 causes a breakdown in vascular integrity throughout the body [16]. We presume the BBB would as well be susceptible to the effects of increased MMP10.

TIMP1 has been shown to be elevated in the CSF of patients with both viral and bacterial meningitis [11], and also in the CSF of patients with Alzheimer’s disease, amyotrophic lateral sclerosis, Parkinson’s disease, and Huntington’s disease [17]. TIMP1 (along with TIMP2) is capable of inhibiting the activities of all known MMPs and is instrumental in maintaining the balance between extracellular matrix deposition and degradation [6]. TIMPs bind to the MMPs forming tight noncovalent complexes [6], [18]. We speculate that TIMP1 levels are increased due to compensatory control mechanisms which ultimately prevent the extracellular matrix from being further disrupted in cALD.

MMP9 was elevated in the plasma of boys with cALD, as well as in the CSF. Elevation of serum MMP9 has been identified in other neurodegenerative disease such as MS where it was found to be elevated in patients with relapsing-remitting MS and other inflammatory neurological diseases as compared to patients with non-inflammatory neurological diseases. It was suggested that the elevated MMP9 serum level may influence the elevations of MMP9 that is observed in the CSF [19]. In our study, the elevated MMP9 plasma levels will probably not serve as a biomarker as many patients had levels of MMP9 similar to that of the controls and the levels not specific enough for cALD, and MMP9 did not correlate with MRI score or neurologic function score (data not shown).

Microglia activation is known to occur in cALD and has been suggested to be partially responsible for the inflammation that causes cALD [3], [20], [21]. Mechanistically, MMPs are probably produced, in part, by locally activated microglia cells and perpetuate the inflammatory process [22]. MMPs have the ability to process interleukin-1 beta into its biologically active form which can then increase BBB permeability [23]. MMPs may also function in an autocrine-type manner whereby they can promote further MMP production by resident or migrating cells, fostering the inflammatory process. Specifically, the MMPs can disrupt the endothelial basal lamina that prevents anchorage of endothelial cells on the extracellular matrix that contributes to BBB breakdown [19].

The increases of MMPs we have found in the CSF of boys with cALD could have several origins including: plasma, choroid plexus, or infiltrating hematopoietic cells, though it likely that all three sources play an intertwined role. Additionally, all the patients in our study had abnormal MRIs at the time of sample collection which begs the question whether elevations in MMP family members occurs before, or as a result of, the damaged BBB. This will be difficult to determine without the prospective sampling of CSF in boys prior to development of cALD. The role of increased MMP2 and MMP9 is an important observation as these MMPs are central to MMP family activation [24]. There is now mounting evidence that activated MMP family members can degrade he the BBB and contribute to neuroinflammation in the context of brain injury neurodegenerative disease, and tumor invasion [25]–[27]. Finally, understanding that there may be a role for MMP family members in the pathology of cALD, may allow the application of MMP inhibitors to be used as potential therapy for boys with cALD; these inhibitors include tetracyclines as well as small molecules directed against MMPs [28], [29]. Whether the BBB disruption can be ameliorated/repaired using MMP inhibitors without undo side effects remains speculative at his point and would require a well-designed clinical trial, though there is some evidence that MMP inhibition can attenuate the inflammatory contribution to cardiovascular disease [28].

It has been previously shown that CSF total protein levels in boys with cALD are elevated [30]. The elevated total protein may lead us to expect that there would be elevation in MMPs, TIMPs, or any CSF-related protein as part of an inflammatory process. High CSF total protein does not undermine the importance of this finding because the proteinases function to degrade matrix in a concentration dependent manner irrespective of the entire total protein makeup (i.e. increased picogram of protein per milliliter increases their activity). The CSF total protein levels also correlated with pre-transplant MRI scores and therefore severity of brain inflammation. Moreover CSF total protein levels correlated with pre-transplant functional scores and post-transplant functional scores. There is precedence for the use of CSF total protein as an indicator of disease progression in other neurodegenerative diseases such as MS [21]. There have not been good indicators of disease progression identified for cALD. Because of the tight correlation of CSF total protein with disease severity, it may be the best biologic identifier of disease progression in boys with cALD.

### Conclusions

We have first identified elevated levels of MMPs and TIMP1 in the CSF of boys with cALD. MMP and TIMP1 levels were directly associated with the severity of neurodegeneration and probably contribute to the disruption in the BBB seen in boys with cALD. This opens the possibility that inhibition of MMPs may provide a method for reducing breakdown of the BBB and neuroinflammation. Finally, CSF total protein level was elevated in boys with cALD, and showed superior correlation to the pre-transplant Loes score as well as the pre- and post- transplant neurologic function score. Although it contains a milieu of different proteins, CSF total protein may be, in fact, the best indicator of disease severity and outcome after HSCT in boys with cALD.
